# A Paradigm Shift from International to Transnational Medical Education

**DOI:** 10.1007/s40670-023-01843-7

**Published:** 2023-07-25

**Authors:** Dhaval Patel, Michael Mullen, Diann S. Eley

**Affiliations:** 1 Ochsner Clinical School, The University of Queensland Faculty of Medicine, 1401 Jefferson Hwy, 70121 Jefferson, LA USA; 2 Academy for Medical Education, Medical School, The University of Queensland, 288 Herston Road, 4006 Brisbane, QLD Australia

**Keywords:** Medical education, Medical curricula, Globalization of medical education, International education exchange, International medical students, International medical graduates

## Abstract

In recent years, the number of medical students seeking international opportunities has grown. To satisfy these demands, collaborative international programs have been developed. However, the benefits of these programs are limited as they employ an international medical education (IME) approach where only the students are exchanged. In this commentary, we discuss the current models of IME and propose a paradigm shift to a transnational approach wherein the student, faculty, and curriculum are exchanged allowing for increased integration and awareness of cultural and educational approaches to treatment that can be retained and incorporated into future practice to advance healthcare across the globe.

## Introduction

The term global health, while seemingly perennial, was first established in the 1990s and became more commonly used by the early 2000s [1]. Stemming from “international health” and even earlier, “tropical medicine,” global health has grown from what was once known as “colonial medicine” [2]. In recent years, the term global health has been demonstrated and tested more than ever as the world united to combat the SARS-CoV-2 pandemic. While this is not an unprecedented event and progress towards unified standards in global health have long been desired, true collaboration and recognition of standards for global health have yet to be fulfilled. Importantly, interest in global health as an academic discipline has grown immensely in recent years, with increasing numbers of medical students expressing a desire to pursue international opportunities [3]. To satisfy these aspirations, a growing number of universities and residency programs are offering global health tracks with international educational opportunities [4–6]. This commentary will work to address the international opportunities that exist for undergraduate medical education and will propose new ideals for progressing towards transnational medical education where the student, teacher, and curricula from multiple nationalities come together to create a medical program fit to address global health needs.

## History of International Medical Education

The pursuit of medical training abroad is not new. In the 1900s, it was a common trend for young physicians to spend 1 or 2 years learning at international institutes. Some notable examples include the cardiothoracic surgeon Michael DeBakey who spent time at the University of Strasbourg and University of Heidelberg, Alton Ochsner Sr. who spent 2 years in Germany and Switzerland, and Harvey Cushing who spent 2 years in Switzerland and the UK [7–9]. In a time when international collaboration and training were virtually impossible without in-person participation, these examples were one-way transactions in which young physicians trained under more experienced physicians to bring home with them newfound expertise and discoveries to advance the medical field in their home countries. These examples were undoubtedly useful in fostering global health initiatives as Michael E. DeBakey, Alton Ochsner Sr., and Harvey Cushing, and others like them, would go on to establish prominent training centers and lead the development of new medical techniques, devices, and treatments.

Since 1971, the University of Virginia neurosurgery program has continued this tradition by sending 5th year residents to be placed in healthcare facilities in Christchurch or Auckland, New Zealand (NZ) instead of reporting to their home base in Virginia. This has allowed them to experience a very different high-performing universal healthcare system while honing surgical skills from a different perspective [10]. More recently, several universities and programs have included international rotations as part of their primary training; however, these experiences are often centered around humanitarian efforts and are for shorter durations than those previously described [11].

## Benefits of International Medical Education

Albeit for a variety of reasons, each of these International Medical Education (IME) opportunities offers a variety of benefits. One major benefit is the exposure to various healthcare systems. There are known problems with medical care in the USA. Possibly the most evident statistic for this is the fact that the USA spends more on healthcare than any country in the world for poorer health outcomes [12]. Part of this problem is due to the lack of universal health coverage (UHC) in the USA. Fundamentally, UHC is the provision of accessible, high-quality healthcare services for all people [13]. The passage of the Affordable Care Act (ACA) pushed the USA closer to achieving UHC by easing access to health insurance; however, over 20 million people remain uninsured with millions more underinsured [14, 15]. This makes the population vulnerable as they are less likely to access healthcare services leading to poorer health outcomes [14, 15]. Most of the top performing health systems such as Sweden, Singapore, Australia, and New Zealand use UHC to ensure access to healthcare services. With a strong push in the USA to move further towards UHC, training in such a system would allow physicians to understand the benefits of UHC while gaining an understanding of how medical service delivery can change.

Hospitals in the USA provide some of the most advanced and specialized care available anywhere in the world. However, this provider model has become overly reliant on technology in lieu of the basic and fundamental physical examination. This trend drives up cost due to unnecessary testing; leads to missed, delayed, or even over diagnosis; and can compromise the patient physician relationship [16]. Other countries such as Ireland, which uses UHC, place much greater emphasis on physical examination skills, using it as the doorway to ordering expensive tests [17]. Furthermore, the age of specialization has led to a noted decrease in skills that are considered mainstream in other health systems. For example, one study noted that general surgery residents from the USA lack skills in basic gynecology, obstetrics, and orthopedics compared to their counterparts from other countries [18]. As healthcare costs in the USA continue to rise, encouraging IME may prove to be beneficial by teaching medical students skills and perspectives that can help reduce healthcare costs while also gaining a bigger picture of healthcare and how it serves the community.

This commentary supports a move towards the provision of more opportunities for global medical education and cultural immersion of students and educators in foreign institutions. However, previous research on the matter has shown that short-term study abroad programs do not engender in students a friendly appreciation or cultural awareness of the host country [19]. It is our belief that to truly grow international mindfulness, exchange of international students, teachers, and curriculum for longer durations and even the entirety of one’s program of study is crucial to fostering the growth of global health perspectives among medical students and educators.

## Shortfalls of International Medical Education

There remain other shortcomings of IME. While IME, post-graduate training, and standardization to the Accreditation Council for Graduate Medical Education International (ACGME-I) guidelines have long been viewed as the standard for progression of global health initiatives [20, 21], the paths by which programs offer IME can take many forms and involve three important variables which are the student, teacher, and curriculum. Commonly, the exchange of any one of these variables is considered IME, the most common of which being a student or teacher studying or practicing at a foreign institution. However, this is a flawed concept as all three variables are crucial for the development of global health initiatives in medical education. Furthermore, these are often one-way tracks with little reciprocity or exchange of learners, educators, and curricula between partner institutions. This has led to an unbalanced distribution of students in what is known as brain-drain from lower-income countries as medical students seek education in higher income countries. Lastly, standardization towards ACGME-I guidelines may invoke colonial-esque implementation of western medical standards that are not suitable for the population they will serve [22].

## Paradigm Shift to Transnational Medical Education

To counteract these paths, and in pursuit of a mission to ensure progression towards global health equity and standardization, perhaps the best place to start is by engaging the medical student in what is known as transnational medical education (TME). First proposed by Dr. Ronald Harden, in this model not just the student, teacher, or curriculum are exchanged between institutions, but all three are exchanged and unified to create a truly collaborative and globally recognized medical education [23]. Simply put, one cannot have a true understanding of global health without international students, international teachers, and international curricula coming together for a unified and standardized TME program. Under this model, TME has been proposed by leading medical educators as the catalyst for the dissemination, collaboration, and standardization of medical education across the world [23].

Perhaps the best example of TME was the International Virtual Medical School (IVIMEDS), which represented a multi-national consortium of medical educators to develop the first transnational medical school. IVIMEDS was composed of 30 international partners from medical universities, educators, and governing boards that sought to standardize medical education through e-learning and virtual reality, which at the time in 2002 was superbly novel and to its own demise potentially ahead of its time [24]. As part of the evaluation process for the IVIMEDS curriculum, partner institutions completed studies investigating the use of IVIMEDS materials as compared to the institutions traditional learning resources. The University of Queensland completed one such study in 2007 which found that students using the IVIMEDS online resources for the cardiovascular module had equal or better assessment outcomes compared to controls [25]. Unfortunately IVIMEDS never reached its full potential as an accredited online medical school.

In today’s age, however, undergraduate medical education has continued to develop online. In particular, pre-clinical training throughout the COVID-19 pandemic was largely replaced by online learning, a true testament to what is now possible through the advent of improved online technologies for medical education [26–28]. While not yet realized, the development of pre-clinical online medical education resources based on the culmination of curriculums across nations should be a primary goal of international medical educators. Through this process, we believe that one day a unified, globally recognized medical curriculum delivered through online resources will be accredited to allow for the education of physicians capable of practicing across the globe.

More recently, traditional brick and mortar medical schools have attempted to establish similar programs. One such example began in 2015 between St. George’s Hospital Medical School (London, UK) and Thomas Jefferson University (Philadelphia, PA). In this program, students spent their early years in the British healthcare system and the latter in the American; however, the program has since been discontinued [29]. The most established and longest running example of TME to date is the transnational partnership between the medical school at the University of Queensland (Brisbane, Australia) and the Ochsner Clinical School (New Orleans, LA). Commencing in 2009, this transnational MD program is not only well established but unique. It is unique because the goal is to educate American citizens across two continents, in two different healthcare systems to practice medicine in the USA and in Australia. American students spend the first 2 years of the MD degree learning in the Australian healthcare system and the latter two in the American healthcare system. This genuine transnational immersion affords a broad exposure of healthcare and medical practice across two continents using a mix of Australian and American curricula, faculty, and students [30].

## Conclusion

It is reasonable to assume that students engaging in TME face similar challenges to those undertaking IME, practicing in a different health system, living in another country, adapting to another culture, and navigating different approaches to learning medicine as well as perspectives on patient care. Nevertheless, these challenges offer a plethora of opportunities for personal and professional growth which may become catalysts for advances in global healthcare. This commentary calls for a paradigm shift that incorporates a transnational approach to medical education. The effort and investment in establishing partnerships that provide a transnational approach are acknowledged but may be justified by the extended knowledge and training of graduates better equipped to solve global healthcare problems. We suggest incorporating transnational experiences in medical education and residency programs on an international scale in order to train physicians who can incorporate global perspectives into their medical practices back in their home country or elsewhere.

## References

[1] Adams LV, Wagner CM, Nutt CT, Binagwaho A (2016). The future of global health education: training for equity in global health. BMC Med Educ.

[2] Farmer PE, Ivers LC (2012). Cholera in Haiti: the equity agenda and the future of tropical medicine. The American Society of Tropical Medicine and Hygiene.

[3] Matheson AI, Pfeiffer J, Walson JL, Holmes K. Sustainability and growth of university global health programs: center for Strategic & International Studies Washington, DC; 2014.

[4] Drain PK, Primack A, Hunt DD, Fawzi WW, Holmes KK, Gardner P (2007). Global health in medical education: a call for more training and opportunities. Acad Med.

[5] Anandaraja N, Hahn S, Hennig N, Murphy R, Ripp J. The design and implementation of a multidisciplinary global health residency track at the Mount Sinai School of Medicine. Acad Med. 2008;83(10). 10.1097/ACM.0b013e3181850a3b 18820521

[6] Drain PK, Holmes KK, Skeff KM, Hall TL, Gardner P. Global health training and international clinical rotations during residency: current status, needs, and opportunities. Acad Med. 2009;84(3). 10.1097/ACM.0b013e3181970a37 PMC3998377 19240438

[7] Salwi S, Chitale RV, Kelly PD (2020). Harvey Cushing's Wanderjahr (1900–1901). World Neurosurg.

[8] Ventura HO (2002). Alton Ochsner, MD: Physician. Ochsner J.

[9] Westaby S. Houston and Oxford: a celebration of international fellowship. Tex Heart Inst J. 2005;32(3):303–17. PMC1336700 16392210

[10] Starke RM, Asthagiri AR, Jane JA, Jane JA (2014). Neurological surgery training abroad as a progression to the final year of training and transition to independent practice. J Grad Med Educ.

[11] Litzelman DK, Gardner A, Einterz RM, Owiti P, Wambui C, Huskins JC (2017). On becoming a global citizen: transformative learning through global health experiences. Ann Glob Health.

[12] Papanicolas I, Woskie LR, Jha AK (2018). Health care spending in the United States and other high-income countries. JAMA.

[13] Universal health coverage (UHC). World Health Organization; 2022 updated 06/29/2023]. Available from: https://www.who.int/news-room/fact-sheets/detail/universal-health-coverage-(uhc). Accessed 23 July 2023.

[14] Envisioning a better U.S. health care system for all: coverage and cost of care. Annals of Internal Medicine. 2020;172(2_Supplement):S7–S32. 10.7326/M19-2415 31958805

[15] Buntin MB. The Affordable Care Act at 10 Years. JAMA Health Forum. 2020;1(7):e200896–e. 10.1001/jamahealthforum.2020.0896 36218705

[16] Faustinella F, Jacobs RJ (2018). The decline of clinical skills: a challenge for medical schools. Int J Med Educ.

[17] Jacobsen AP, Khiew YC, Murphy SP, Lane CM, Garibaldi BT (2020). The modern physical exam – a transatlantic perspective from the resident level. Teach Learn Med.

[18] Lin Y, Dahm JS, Kushner AL, Lawrence JP, Trelles M, Dominguez LB (2018). Are American surgical residents prepared for humanitarian deployment?: A comparative analysis of resident and humanitarian case logs. World J Surg.

[19] Teichler U (2003). Mutual recognition and credit transfer in europe: experiences and problems. J Stud Int Educ.

[20] Day SH, Nasca TJ (2019). ACGME International: the first 10 years. J Grad Med Educ.

[21] Archuleta S, Chew N, Ibrahim H (2019). The value of international research and learning in graduate medical education. J Grad Med Educ.

[22] Karle H, Christensen L, Gordon D, Nystrup J (2008). Neo-colonialism versus sound globalisation policy in medical education. Med Educ.

[23] Harden RM (2006). International medical education and future directions: a global perspective. Acad Med.

[24] Harden RM, Hart IR (2002). An international virtual medical school (IVIMEDS): the future for medical education?. Med Teach.

[25] Régo P, Ozolins I (2007). IVIMEDS: a short report on an evaluation of the cardiovascular system learning module. Med Teach.

[26] Binks AP, LeClair RJ, Willey JM, Brenner JM, Pickering JD, Moore JS (2021). Changing medical education, overnight: the curricular response to COVID-19 of nine medical schools. Teach Learn Med.

[27] Rose S (2020). Medical student education in the time of COVID-19. JAMA.

[28] Stoehr F, Müller L, Brady A, Trilla A, Mähringer-Kunz A, Hahn F (2021). How COVID-19 kick-started online learning in medical education-The DigiMed study. PLoS ONE.

[29] Thomas Jefferson University [Internet]. News & Events. Philadelphia: TJU; January 21st, 2015. Unique Transatlantic Partnership to Train Doctors for Global Health Challenges. Available from: https://www.jefferson.edu/university/news/2015/01/21/st-georges-thomas-jefferson-university-partnership.html. Accessed 23 July 2023.

[30] Deichmann RE, Alder L, Seoane L, Pinsky WW, Denton GD (2016). Initial match rates of an innovative international partnership: the Ochsner Clinical School experience. Ochsner J.

